# Supplementary material for: Climate Variations in the Low-Latitude Plateau Contribute to Different Sugarcane (Saccharum spp.) Yields and Sugar Contents in China
Source: Plants (Basel). 2023 Jul 21;12(14):2712. doi: 10.3390/plants12142712 (PMC10385836; doi:10.3390/plants12142712)
Supplement: Supplementary file 1 [file plants-12-02712-s001.zip › plants-2461649-supplementary.pdf]

Table s1 Summary of average (± standard error) annual AAT, ARH, AAR, AAS, yield, and sucrose content of the sites across all years.

Site	Site-year	AAT/℃		ARH/%		AAR/mm		AAS/h		Yield/t		Sucrose content/%	
		Mean ± standard error	CV/%	Mean ± standard error	CV	Mean ± standard error	CV/%	Mean ± standard error	CV/%	Mean ± standard error	CV%	Mean ± standard error	CV
S1	11	18.21±0.28	1.52	79.88±1.7	2.13	137.06±16.72	12.20	155.42±11.44	7.36	3.87±0.37	9.68	14.71±0.42	2.83
S2	11	17.16±0.31	1.83	71.82±2.32	3.23	98.30±10.41	10.59	172.09±23.05	13.39	3.60±0.53	14.35	14.32±0.61	4.26
S3	6	20.38±0.54	2.67	76.66±2.34	3.05	101.99±18.79	18.43	140.99±11.75	8.33	3.02±0.43	14.30	13.66±0.81	5.94
S4	11	19.90±0.32	1.58	71.91±2.33	3.24	101.50±15.50	15.27	185.33±10.67	5.76	3.70±0.24	6.36	14.48±0.31	2.12
S5	10	17.65±0.46	2.59	76.15±2.12	2.78	82.64±14.40	17.43	129.67±6.73	16.41	4.31±1.01	23.65	13.10±1.03	7.84
S6	10	20.85±0.59	2.82	72.15±1.59	2.2	68.60±9.99	14.56	168.22±15.80	9.39	3.44±0.64	18.52	14.61±0.47	4.71
S7	3	18.37		82.07		187.58		162.2		4.61		14.41	
S8	9	19.26±0.63	3.28	72.94±2.53	3.47	86.65±17.45	19.69	173.48±21.37	12.32	3.48±0.57	16.39	13.97±0.57	4.06
S9	9	20.93±0.30	1.42	73.37±2.93	4.00	98.24±12.18	12.40	181.29±15.77	8.70	2.66±0.74	20.23	14.26±0.48	3.34
S10	10	20.19±0.29	1.43	75.90±2.51	3.31	135.67±13.60	10.02	186.00±12.71	6.83	3.12±0.40	12.82	14.21±0.41	2.91
S11	10	18.66±0.20	1.05	76.77±2.13	2.78	104.68±17.72	16.93	195.51±18.25	9.34	4.21±0.57	13.55	13.90±0.60	4.32
S12	11	18.23±0.18	0.98	68.17±2.27	3.33	89.14±11.48	12.88	197.79±16.38	8.28	3.42±0.28	8.05	14.53±0.37	2.56
S13	11	19.58±0.19	0.98	76.37±2.50	3.28	113.46±17.15	15.12	184.32±20.30	11.01	4.75±0.52	11.00	14.45±0.44	3.02
S14	10	20.32±0.26	1.26	75.27±1.69	2.25	124.48±16.20	13.01	174.18±5.50	3.15	4.08±0.41	10.05	14.15±0.37	2.58
S15	11	19.46±0.43	2.23	77.25±2.63	3.41	108.18±9.86	9.11	162.83±9.85	6.05	4.09±0.57	13.98	14.03±2.85	2.85
S16	10	22.04±0.32	1.47	79.96±1.06	1.33	128.59±14.52	11.29	165.78±17.98	10.85	3.02±0.35	11.54	14.52±0.39	2.66
S17	11	20.39±0.20	0.96	81.64±0.82	1.01	108.44±14.54	13.41	182.17±14.53	7.98	4.01±0.61	15.33	15.09±1.03	6.8
S18	9	21.03±0.28	1.34	74.39±2.92	3.93	111.43±11.75	10.55	192.57±15.86	8.24	4.89±0.32	6.62	15.11±0.41	2.7
S19	9	18.05±0.40	2.23	66.06±2.36	3.58	73.31±11.50	15.69	193.80±14.47	7.47	3.13±0.51	16.18	1487±0.67	4.49
S20	10	18.42±0.57	3.11	71.48±2.22	3.11	69.03±11.03	15.97	179.56±11.33	6.31	3.91±1.29	32.95	14.27±0.68	4.75
S21	11	20.18±0.25	1.25	71.54±2.92	4.08	78.77±10.48	13.30	208.33±13.90	6.67	4.01±0.30	7.39	14.56±0.26	1.77
S22	3	18.63		74.00		79.85		176.86		4.00		13.47	
S23	11	19.61±0.25	12.6	77.64±1.52	16.6	153.19±9.00	5.87	172.74±10.64	6.16	3.30±1.31	39.59	13.96±0.49	3.48
S24	11	20.37±0.20	0.99	74.15±2.21	2.98	114.78±20.09	17.51	196.07±15.72	8.02	4.62±0.33	7.22	14.56±0.53	3.67
S25	11	20.55±0.38	1.86	68.06±2.36	3.46	67.98±15.41	22.67	194.90±18.31	9.39	3.43±0.62	18.16	14.27±0.55	3.86
S26	10	20.03±0.36	1.80	77.33±1.45	1.88	128.54±16.52	12.85	169.65±11.48	6.77	3.36±0.29	8.62	14.64±0.40	2.71

Footnote: AAT, Average air temperature; ARH, Average relative humidity; ARA, Average rainfall amount; ASD, Average sunshine duration.

Table s2 Summary of average (± standard error) AAT, ARH, AAR, AAS, yield, and sucrose content of the sites across all years.

Crop /Year	Year-Site	AAT/℃		ARH/%		AAR/mm		AAS/h		Yield/t		Sucrose content/%	
		Mean ± standard error	CV/%	Mean ± standard error	CV	Mean ± standard error	CV/%	Mean ± standard error	CV/%	Mean ± standard error	CV/%	Mean ± standard error	CV
2005/2006	25	19.63±1.11	5.65	74.58±4.45	5.96	97.78±31.27	31.98	173.91±19.95	11.47	3.75±0.71	19.43	14.05±0.80	5.72
2006/2007	24	19.45±1.19	6.11	74.82±3.85	5.14	107.51±31.99	29.76	175.26±22.49	12.83	3.68±1.31	3.36	14.03±0.49	3.50
2007/2008	26	19.07±1.24	6.52	74.98±3.66	4.88	114.63±27.23	23.76	167.89±19.29	11.49	3.87±0.72	18.63	14.05±0.60	4.30
2008/2009	26	19.37±1.17	6.04	74.80±4.23	5.65	110.67±30.53	27.58	161.78±22.46	13.88	3.78±0.60	15.76	14.60±0.54	3.70
2009/2010	23	19.65±1.15	5.85	71.69±5.73	7.99	90.93±30.14	33.15	178.30±22.38	12.55	3.94±1.32	33.43	14.80±0.44	2.96
2010/2011	25	19.76±1.20	6.07	74.22±5.09	6.86	107.66±36.91	34.29	172.32±25.85	15.00	3.54±0.77	21.82	14.21±0.56	3..96
2011/2012	24	19.53±1.12	5.74	75.95±5.00	6.59	104.87±30.74	29.31	180.07±25.39	14.10	3.57±0.75	21.13	14.42±0.63	4.40
2012/2013	24	19.96±1.26	6.29	73.45±5.13	6.98	95.00±32.53	34.24	188.81±22.93	12.14	3.53±0.73	20.74	14.52±0.91	6.30
2013/2014	25	19.41±1.19	6.15	73.66±5.04	6.85	111.67±34.35	30.76	187.91±17.21	9.16	3.76±0.71	18.9	14.00±1.10	7.85
2014/2015	24	19.81±5.98	5.98	74.64±5.36	7.18	108.99±33.84	31.05	192.02±21.06	10.97	3.78±0.74	19.51	14.27±0.64	4.48
2015/2016	26	19.71±1.19	6.06	76.07±4.52	5.95	109.33±31.92	29.19	173.24±13.67	7.89	3.81±0.69	18.10	14.16±0.83	5.87

Footnote: AAT, Average air temperature; ARH, Average relative humidity; ARA, Average rainfall amount; ASD, Average sunshine duration.
